# Foveal macular pigment dip in offspring of age-related macular degeneration patients is inversely associated with omega-3 index

**DOI:** 10.1186/s12886-020-01742-0

**Published:** 2020-12-02

**Authors:** Grant A. Rutledge, Steven G. Pratt, Stuart P. Richer, Byki Huntjens, C. Blake Perry, Gunilla Pratt, Carla Podella

**Affiliations:** 1 Scripps Health/Scripps Memorial Hospital/Scripps Mericos Eye Institute - Scripps Clinical Research Service, La Jolla, CA USA; 2 Department of Ecology and Evolutionary Biology, University of California, Irvine, CA USA; 3 USDA Human Nutrition Research Center on Aging, Boston, MA USA; 4 Eye Clinics, Captain James A. Lovell Federal Health Care Center, North Chicago, IL USA; 5 Centre for Applied Vision Research, Division of Optometry and Vision Sciences, City, University of London, London, UK

**Keywords:** Macular pigment (MP), Macular pigment optical density (MPOD), Foveal macular pigment dip (FMPD), Lutein, Zeaxanthin, Eicosapentaenoic acid (EPA), Docosahexaenoic acid (DHA), Omega-3 index

## Abstract

**Background:**

Offspring of parent(s) with age-related macular degeneration (AMD) have a 45% lifetime risk of developing the disease. High foveal macular pigment optical density (MPOD) is protective, whereas individuals with a “foveal macular pigment dip” (FMPD) are at increased risk. Shortage of the dietary carotenoids lutein, zeaxanthin as well as fish consumption are reported AMD risk factors. This Early Biomarkers of AMD (EBAMD) study evaluates serum factors that protect foveal MPOD architecture in Caucasian offspring of parent(s) with AMD.

**Methods:**

*N* = 130 subjects [mean (SD) age 62.8 (8.6) years; 36/94 male/female] were recruited from Scripps Health/ Scripps Memorial Hospital/ Scripps Mericos Eye Institute between 2012 and 2017. Macula pigment 3D topography was evaluated using specular reflectance. Buccal genetic cheek swab, circulating serum dietary carotenoids and long-term RBC omega-3 fatty acid status, as well as common secondary clinical structural and vision function parameters were obtained.

**Results:**

41 % of offspring of AMD parent(s) presented with FMPD. These offspring were about 4 years younger than those without FMPD (controls; *P* = 0.012) and had thinner foveas (*P* = 0.010). There were no differences in gender, BMI, % body fat, visual acuity or contrast sensitivity between those with and without FMPD. % RBC membrane docosahexaenoic acid (DHA) was reduced in FMPD offspring vs. control offspring (*P* = 0.04). The Omega-3 Index was significantly decreased in the FMPD group (*P* = 0.03).

**Conclusions:**

The percentage of FMPD in AMD offspring is nearly twice that reported for the general population in the scientific literature. Offspring presenting FMPD had similar AMD genetic risk, but significantly reduced % RBC membrane omega-3 fatty acids and thinner foveas compared with those without FMPD. Our data supports the importance of ‘essential fatty’ acids as an independent AMD risk factor.

## Background

Age related macular degeneration (AMD) causes progressive loss of vision in older adults [1] and is responsible for significant visual impairment in the United States [2]. Genetic studies show hereditary susceptibility to developing AMD [3–5], whereby monozygotic twin studies have identified an increased risk for developing AMD in individuals whose identical twin has AMD even when environmental factors are not shared [6]. Other studies support this shared genetic predisposition for AMD among siblings and twins [7, 8].

Human macular pigment consists of the two dietary xanthophyll carotenoids, lutein and zeaxanthin, as well as lutein’s metabolite meso-zeaxanthin [9, 10]. Reduced central macular pigment optical density (MPOD) is often associated with major AMD risks including increased age, family history, smoking [11], female gender [12], light iris color [13], and inflammatory conditions such as diabetes [14]. Healthy appearing retinas without AMD but predisposed to the disease due to advance AMD in the fellow eye, presented significantly less MPOD than aged-matched controls [10]. Although MPOD was not measured in the Age-related Eye Disease Study II (AREDS II), repletion of lutein and zeaxanthin in high risk AMD patients was associated with a 26% AMD risk reduction in the low dietary carotenoid intake group, suggesting a causal role within a recent review of macular pigment [15]. In addition, macular pigment architecture most commonly declines exponentially from the foveolar [16–18]. Atypical MPOD spatial profiles have been observed in approximately 20% of subjects containing a secondary pigment peak/ring [19–22] or central dip [23–25] here collectively termed the “foveal macular pigment dip” (FMPD). Increased prevalence of atypical central dip profiles is a further risk factor for AMD, and has been associated with age [24], smoking [24], certain ethnicities [25], and intake of dietary carotenoids [26].

AMD risk variants in the complement system point to the important role of the immune response and inflammation in the pathogenesis of AMD [27]. An established dietary risk factor for the development of AMD is omega-3 fatty acid intake, with emerging emphasis upon the docosahexaenoic (DHA) fraction [28–30]. A systematic literature review determined that dietary omega-3 fatty acids are associated with lower risk for developing AMD [31]. It is thought that higher fish intake is associated with lower risk for developing AMD, in part because of its high concentrations of DHA [32]. Furthermore, DHA accounts for approximately 50% of the polyunsaturated fatty acids (PUFAs) of cell membranes in the central nervous system with the highest concentrations within photoreceptor outer segments and synapses [33].

The primary aim of the early biomarkers of AMD study (EBAMD) is to investigate the prevalence of FMPD amongst healthy but high-risk AMD offspring. Secondly, EBAMD evaluates its association with genetic risk, carotenoid and omega-3 fatty acid status, and other subclinical biomarkers.

## Methods

### Recruitment

Informed consent following the tenets of the Declaration of Helsinki were obtained from a staggered recruitment registry of *n* = 140 non-smoking offspring of AMD parent(s) in the well-educated, well nourished, active and affluent Caucasian population of La Jolla, CA. The 5-year study commenced on 01/01/2012 following Scripps Health IRB approval (11–5677). Subjects were recruited from Scripps Health/ Scripps Memorial Hospital/ Scripps Mericos Eye Institute. Individuals interested in participating in the study were asked to participate in a free screening visit/eye exam conducted by the study physician (SGP). Subjects meeting all inclusion criteria after the screening visit were asked to participate in the study and scheduled for their study visit.

### Inclusion criteria

1) Age > 40 years; 2) no history of cigarette smoking; 3) No visible AMD pathology defined by AREDS [34] or confounding ocular/ systemic disease; 4) Mother and/or father with AMD diagnosed by an ophthalmologist. 5) Patients were free of other ocular and systemic diseases that could affect MPOD, ocular structure and visual function as determined by an ophthalmologist (SGP).

### MPOD and dermal carotenoids

Objective 3D Specular reflectance MPOD topography of peak and integrated volume were determined by the method of specular reflectance using an ARIS (Automated Retinal Imaging System, Visual Pathways, Inc., Prescott, AZ) [35]. The measurement involves capturing a specular reflectance single wavelength 500 nm image, through a dilated pupil at 2 retinal locations: the fovea having maximum MPOD and a peripheral retinal location where MPOD is minimal. Peak subjective heterochromic flicker photometric 1-degree MPOD using QuantifEye® (ZeaVision, Chesterfield, MO) was also acquired.

**Fig. 1:**
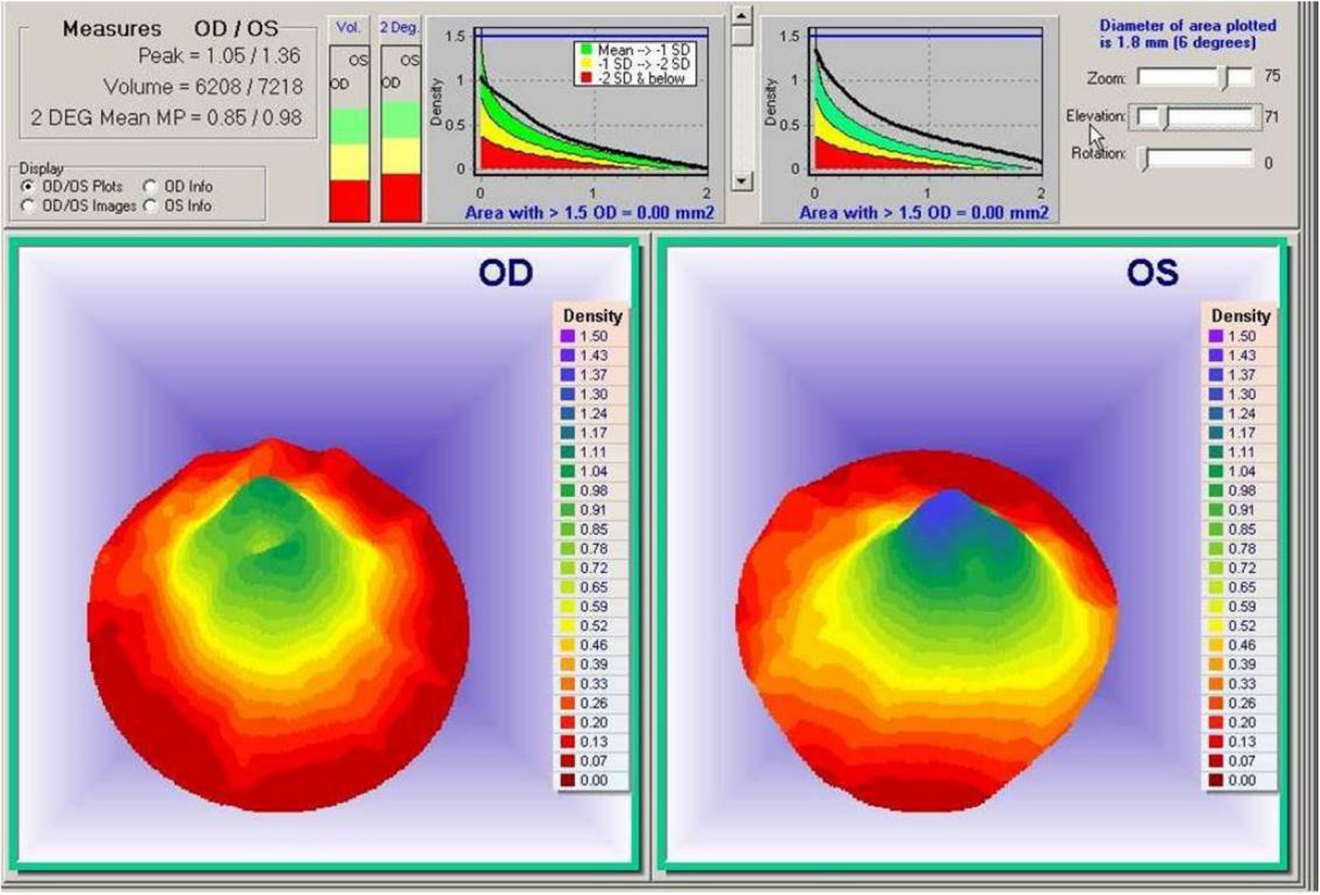
Baseline ARIS specular reflectance images showing the macula pigment foveal dip phenomenon in subject #22, female, age 61. The OD image shows a foveal macular pigment dip (FMPD) while OS shows a normal macula pigment distribution

The ARIS Macurate™ Macular Pigment software module displays the 7-degree color coded 3D in vivo representation of MPOD architecture, quantifying the 1-degree peak, 2-degree peak and volume of retinal foveal carotenoid pigmentation using single wavelength auto-reflectance [36–38] (Fig. 1). Unidentifiable images were removed from the data set, *n* = 10 (BH). We defined the MPOD spatial profile as a central dip if visually present and at least one coefficient of repeatability (CoR; i.e., the average within-subject SD) below the MPOD measurement at 2 degrees). The CoR of the ARIS was found to be 0.17 density units (du) following repeated MPOD measurements in 11 volunteers.

Peak subjective heterochromic flicker photometric MPOD at 1 degree was also determined using the QuantifEye® (ZeaVision, Chesterfield, MO) instrument. Palm skin carotenoids, a measure of systemic carotenoid intake, was determined using the resonant Raman spectroscopic BioPhotonic® Scanner (Pharmanex, Inc., Provo, UT) [39].

### Foveal retinal thickness, visual acuity and contrast sensitivity

Spectral domain optical coherence tomography (SD-OCT) scans, for foveal retinal thickness (cRT) through dilated pupils, was accomplished with the Topcon Spectral Domain 100 (Oakland, NJ) in a subset (*n* = 72) of offspring. Snellen visual acuity through best spectacle correction was recorded and converted to decimal format. Photopic contrast sensitivity at 5 spatial frequencies was evaluated using a Stereo Optical Functional Vision Analyzer (Stereo Optical, Chicago, IL, USA) and the Area Under Curve (AUC) calculated as described in our previous studies [36, 40].

### Serum carotenoids, RBC omega-3 fatty acids and Spectracell®

Serum carotenoids (lutein and zeaxanthin) from 126 participants were determined by Pennington Biomedical Research Center (www.pbrc.edu). Volunteers were grouped according to low (< 2.9 μg/dL) or high serum Z (≥2.9 μg/dL) since the HPLC column used to measure Z was not sensitive to values lower than 2.9 μg/dL. The HS-Omega-3 Index by OmegaQuant (developed by True Health Diagnostics, Frisco, TX) was used to determine the red blood cell content of the following fatty acids: Omega-3 Total, Alpha-Linolenic Acid (ALA), Docosapentaenoic (DPA), Eicosapentaenoic (EPA), Docosahexaenoic (DHA), HS-Omega-3 Index (RBC EPA + DHA), Omega-6 Total, Arachidonic (AA), Linoleic (LA), Cis-Monounsaturated Total, Saturated Total, and Trans-Fat Index. SpectraCell® Laboratories (Houston, TX; www.spectracell.com) testing was ascertained to gauge further insight with respect to nutrient factors affecting the RBC omega-3 results [41].

### Genetics

A subgroup of participants (*n* = 99) were buccal cheek-swabbed, segregated into 5 risk categories and categorized for total risk & lifetime risk using a 5 genotype CFH, C3, ARMS2 & MT-ND2 8 SNP DNA array (www.njlabs.com) [42]. We also evaluated the SNP (single nucleotide polymorphisms) methylenetetrahydrofolate reductase (MTHFR) C677T and A1298C for folic acid, because of their potential synergistic relationship to DHA status [43].

### Statistics

All statistical analyses were performed in SPSS version 25.0 for Windows (SPSS, Inc., Chicago, IL). Independent Student’s *t*-tests analyzed the differences between the MPOD spatial profile groups (with and without foveal dip) and those with or without measurable carotenoids in the serum, whereas an ANCOVA was used to correct for age where appropriate. Kolmogorov-Smirnov tests revealed a significant deviation from a normal distribution for all variables except age, % body fat, MPOD, cRT, contrast (not contact) sensitivity, DHA, Omega-3 index, skin carotenoids, zinc, homocysteine, HDL cholesterol, HDL 2b and COQ10. The Pearson χ2 test and Mann-Whitney *U* test assessed any difference between categories and groups that showed an abnormal distribution. Preliminary analysis ensured no violation of assumptions of normality, or high correlation between the independent variables. Binomial logistic regression ascertained the effects of MPOD peak and volume, EPA, DHA, and Omega-3 index against FMPD presence. We also applied the binomial logistic regression analysis to further ascertain the effects of Holman Omega-3 Index modifiers: COQ10, anti-inflammatory Spectracell® (Houston, TX) serum calcitriol 1, 25 OH Vitamin D3 white blood cell (WBC) activity, total high-density lipoprotein (HDL), HDL 2b, hs-CRP, and LP(a) against FMPD presence. Statistical significance was accepted at the 95% confidence level (*P* < 0.05).

## Results

### Demographic, genetic, macular, and skin carotenoid pigmentation

Table 1 displays characteristics of the offspring of AMD patients with and without a FMPD. Population peak MPOD significantly decreased with age (Spearman’s rho r_s_ (128) = − 0.519, *P* < 0.0001) as did MPOD volume (Spearman’s rho r_s_ (127) = − 0.524, *P* < 0.0001), indicating 27% of the variance in both parameters was explained by age. There was no significant relationship between central 1-degree foveal MP and the MP distribution integrated area, using either specular reflectance or QuantifEye® heterochromic flicker photometry.

**Table 1:** EBAMD Subject characteristics: FMPD (Foveal Macular Pigment Dip) versus No Dip (corrected for age). Mean ± SD data is presented unless otherwise stated. Statistical significance is presented in bold

Variable	Total	MPOD spatial profile: FMPD	MPOD spatial profile: No Dip	P
**Offspring subjects (%)**	130 (100%)	53 (41%)	77 (59%)	
**Age (years)**	62.8 ± 8.6	60.6 ± 8.0	64.4 ± 8.7	**0.012**
**Gender**	36 (28%):	11 (21%):	25 (32%):	0.14
Male: Female (%)	94 (72%)	42 (79%)	52 (68%)	
**% Body Fat** (bioelectric impedance)	32.6 ± 7.8	32.6 ± 7.7	32.5 ± 8.0	0.92
**Body Mass Index** (kg/m^2^)	25.4 ± 4.8	25.4 ± 5.3	25.5 ± 4.5	0.49
**ARIS MP Peak** (du)	0.80 ± 0.21	0.85 ± 0.20	0.77 ± 0.21	0.39
**ARIS MP Volume**	3963 ± 1222	4310 ± 1156	3723 ± 1217	0.10
**Central retinal thickness (**μm**)** (*n* = 72)	238 ± 21.0	232 ± 17.8	243 ± 22.2	**0.010**
**Visual acuity** (converted to decimals)	0.92 ± 0.22	0.91 ± 0.22	0.93 ± 0.21	0.61
**Contrast sensitivity** (AUC)	362 ± 109	362 ± 104	361 ± 114	0.94
**DHA** (%)	4.94 ± 1.42	4.62 ± 1.36	5.15 ± 1.42	**0.040**
**EPA** (%)	1.07 ± 0.68	0.92 ± 0.559	1.16 ± 0.744	0.10
**HS-n3 index** (%)	6.03 ± 1.99	5.55 ± 1.80	6.34 ± 2.05	**0.029**
**Serum L** (μg/dL)	24.8 ± 15.7	23.7 ± 13.1	25.6 ± 17.3	0.98
**Serum Z** (μg/dL)	6.89 ± 6.12	6.34 ± 5.41	7.28 ± 6.59	0.34
**L: Z ratio**	4.45 ± 1.84	4.75 ± 2.20	4.24 ± 1.52	0.31
**Skin carotenoids**	34,525 ± 13,970	33,528 ± 14,331	35,225 ± 13,766	0.50
**Folic acid mutations**				
Negative: Heterozygous: Homozygous				
**C677T**	57 (46%): 47 (38%): 20 (16%)	26 (52%): 18 (36%): 6 (12%)	31 (42%): 29 (39%): 14 (19%)	0.44
**AI298C**	45 (36%): 57 (46%): 22 (18%)	16 (32%): 23 (46%): 11 (22%)	29 (39%): 34 (46%): 11 (15%)	0.53

*Du* Density units, *AUC* Area under the curve, *MPOD* Macular pigment optical density, *FMPD* Foveal macular pigment dip, *MP* Macular pigment, *DHA* Docosahexaenoic acid, *EPA* Eicosapentaenoic acid, *Serum L* Serum Lutein, *Serum Z* Serum Zeaxanthin

FMPD was common in AMD offspring as *n* = 53 (41% of offspring) presented this phenomenon. 62% (*n* = 20) displayed the phenomenon in one eye and 38% (*n* = 33) displayed a foveal dip in both retinas, with no gender, BMI or %-age body fat effect. Paradoxically, younger age was significantly associated with an FMPD central dip (*P* = 0.012), and FMPD offspring also had denser average bi-retinal peak MPOD (0.85 ± 0.20 du), compared with those without FMPD (0.77 ± 0.21 du). This difference, however, was not significant after correction for age (F (1, 125) = 0.757, *P* = 0.39, η_p_^2^ = 0.006). FMPD offspring also had denser average bi-retinal MPOD volume (4310 ± 1156 du) compared to those without FMPD (3723 ± 1217 du), however once again corrected for age, this seemingly compensatory increase in non-foveal retinal pigmentation was non-significant (F (1, 124) = 2.84, *P* = 0.095, η_p_^2^ = 0.022).

A comprehensive ophthalmological evaluation was completed on all patients by the same examiner (SGP) revealing no clinically significant drusen or retinal pigment epithelium changes. All patients had a best corrected vision of 20/25 or better in both eyes. SD-OCT structural data was determined on a population subset (*n* = 72). After correcting for age, central retinal thickness at the fovea (cRT) was thinner where FMPD was observed (232 ± 17.8 μm), versus without a MPOD dip (243 ± 22.2 μm; F (1,69) = 7.09, *P* = 0.01). Neither visual acuity nor contrast sensitivity discriminated between the two offspring groups (*n* = 130, *P* = 0.61 and *P* = 0.94; Table 1).

Population skin carotenoids scores, a surrogate measure of systemic carotenoids, were 34,525 ± 13,970 with no correlation to age (Pearson r = 0.12; *P* = 0.18). Skin carotenoids were lower in those with a FMPD (33,528 ± 14,331 units) versus those without (35,225 ± 13,766 units) but not statistically significant (*P* = 0.50). Serum L, Z and the L:Z ratio are shown in Table 1. Although not significant, serum L and Z were both decreased and the L:Z ratio was increased when a FMPD was present, compared to those without a central dip (*P* = 0.31).

A Chi-square test for independence was performed to investigate the relationship between FMPD and 5 prominent DNA risk categories. Risk categories 1 and 2 to 4 were compared to prevent violation of the frequency assumption (count > 10), as categories 3 and 5 were infrequent. The proportion of participants showing an FMPD and higher risk genetic scores did not significantly differ from those without a dip [χ2(1) = 0.029, *P* = 0.87]. MTHFR SNPs C677T and A1298C showed no significant difference in those presenting with FMPD compared to controls (*P* = 0.44 and *P* = 0.53, respectively) (Table 1).

### Omega-3 index, DHA, and EPA

The RBC omega-3 fatty acid status was different in FMPD offspring versus those without a central dip (Table 1). RBC content of docosahexaenoic acid (DHA) was reduced in FMPD offspring vs. controls [4.62% (1.36%) vs. 5.15% (1.42%); *P* = 0.04]. RBC Eicosapentaenoic acid (EPA) also trended lower in FMPD subjects vs. controls [0.92% (0.56%) vs. 1.16% (0.74%); *P* = 0.10], although not significantly. However, the Omega-3 Index (RBC EPA + DHA) was significantly decreased in FMPD offspring [5.55% (1.80%) vs. 6.34% (2.05%); *P* = 0.03].

### Predictors of MPOD foveal dip status

A binomial logistic regression was performed to ascertain the effects of MPOD peak and volume, EPA, DHA, Omega-3 index on the likelihood of offspring having a FMPD. The logistic regression model was statistically significant [χ2(5) = 11.81, *P* = 0.038]. The model explained 12.8% (Nagelkerke R^2^) of the variance in FMPD and correctly classified 68% of cases. *Specificity* of our model was 86% with a *positive predictive value* of 67%. We applied the binomial regression analysis to further ascertain the effects of Holman Omega-3 index modifiers: CoQ10, anti-inflammatory Spectracell® (Houston, TX) serum calcitriol 1, 25 OH Vitamin D3 white blood cell (WBC) activity, Total High-density lipoprotein (HDL), HDL 2b, hs-CRP, and LP(a) on the likelihood that participants presented with a FMPD. The logistic regression model was highly suggestive, but not statistically significant [χ2(6) =11.952, *P* = 0.063].

### Serum zeaxanthin

40% of subjects presented with low serum Z levels (< 2.9 μg/dL). FMPD offspring exhibiting low serum Z (44%) did not statistically differ compared to controls (37%; *P* = 0.49). We further investigated the effect of low serum Z on all our serum nutritional biomarkers because of zeaxanthin’s central foveola location. Independent t-tests and Mann-Whitney U test results are shown in Table 2. Those subjects with low serum Z showed significantly higher contrast sensitivity (*P* = 0.049), but significantly decreased RBC DHA (*P* = 0.00034) and EPA (*P* = 0.041), decreased RBC Omega 3 (*P* = 0.0025), decreased RBC Omega-3 index (*P* = 0.0011), increased Spectracell (1,25 hydroxy-vitamin D) calcitriol anti-inflammatory activity (*P* = 0.037), fewer HDL 2b molecules (*P* = 0.018) and increased Chromium (*P* = 0.014) compared to subjects whose serum zeaxanthin was ≥2.9 μg/dL.

**Table 2:** Independent t-test and non-parametric Mann-Whitney^†^ test analyses of differences in nutritional biomarkers for those presenting low (< 2.9 μg/dL) versus high (≥ 2.9 μg/dL) serum zeaxanthin. Mean ± SD data is presented. Statistical significance is presented in bold

Variable	Serum Z (< 2.9 μg/dL)	Serum Z (≥2.9 μg/dL)	P
**Offspring subjects (%)**	50 (40%)	76 (60%)	
**Age (years)**	61.4 ± 9.1	63.7 ± 8.3	0.15
**% Body Fat measurement**	33.0 ± 8.5	32.2 ± 7.5	0.62
**Body Mass Index** (kg/m^2^)	26.5 ± 5.8	24.7 ± 4.1	0.11^†^
**ARIS MP Peak (du)**	0.80 ± 0.22	0.81 ± 0.20	0.95
**ARIS MP Volume**	3949 ± 1310	4005 ± 1162	0.81
**Central retinal thickness (μm)** (*n* = 68)	239 ± 22	237 ± 19	0.82
**Visual acuity** (decimals)	0.91 ± 0.22	0.92 ± 0.22	0.77^†^
**Contrast sensitivity** (AUC)	383 ± 100	344 ± 113	**0.049**
**DHA** (%)	4.36 ± 1.35	5.31 ± 1.37	**0.00034**
**EPA** (%)	0.92 ± 0.58	1.17 ± 0.74	**0.041**^**†**^
**HS-n3 index**	5.30 ± 1.84	6.52 ± 1.99	**0.0011**
**Skin carotenoids**	33,132 ± 12,334	35,763 ± 14,896	0.31
**Homocysteine** (μmol/L)	9.7 ± 2.6	9.9 ± 2.5	0.71
**Zinc**	40.7 ± 6.7	41.8 ± 5.1	0.303
**Copper**	52.0 ± 5.3	52.0 ± 5.3	0.91^†^
**Vitamin**			
**B1**	91.2 ± 8.2	92.6 ± 7.9	0.39^†^
**B3**	92.0 ± 6.1	92.4 ± 6.9	0.72^†^
**B6**	63.0 ± 7.2	65.5 ± 5.6	0.079^†^
**B9 (folate)**	40.54 ± 5.1	41.45 ± 6.2	0.19^†^
**B12**	18.8 ± 3.9	18.6 ± 4.5	0.87^†^
**D3** (1,25 WBC Calcitriol Activity)	64.9 ± 10.8	61.878 ± 8.9	**0.037**^**†**^
**Omega 3**	8.1 ± 2.2	9.5 ± 2.4	**0.0025**^**†**^
**Insulin (**μlU/mL)	46.9 ± 8.2	46.6 ± 8.0	0.81^†^
**Chromium**	46.8 ± 4.7	44.8 ± 5.1	**0.014**^**†**^
**HDL cholesterol** (nmol/L)	64.3 ± 15.3	70.8 ± 19.5	0.0556
**Total HDL Particles** (nmol/L)	8674 ± 1016	8379 ± 1408	0.099^†^
**HDL 2b** (nmol/L)	2393 ± 642	2701 ± 714	**0.0182**
**hs-CRP** (mg/L)	1.78 ± 2.11	2.05 ± 2.74	**0.64**^†^
**COQ10**	91.2 ± 4.23	90.9 ± 4.15	0.71
**LP(a) (mg/dL)**	19.9 ± 24.1	30.2 ± 41.6	0.34^†^

*Serum Z* Serum Zeaxanthin, *MP* Macular Pigment, *DHA* Docosahexaenoic acid, *EPA* Eicosapentaenoic acid, *HDL* High-density lipoprotein, *hs-CRP* High Sensitivity C-Reactive Protein, *LP* Lipoprotein

^†^signifies that a Man-Whitney test was used to obtain the corresponding p-value

## Discussion

EBAMD is a preventative medicine analysis of genetic, nutritional and subclinical risk factors for developing AMD, given a parental AMD history [32]. MPOD dip prevalence in at least one eye of the offspring of AMD parent(s) was surprisingly high at 41%, double that reported within the general Caucasian population [23, 25, 32]. This was unexpected since our population already excluded offspring more likely to have atypical profiles (i.e. foveal macula pigment dip) such as non-Caucasians with larger concentrations of skin pigments [25, 44, 45] smokers [17] and diseased retinas (AMD patients) [22]. Consistent with the MPOD literature [10, 46, 47], we found significantly decreased MPOD with age. AMD genetic risk test failed to reflect a difference, suggesting that additional SNPs, multiple systemic alleles or environmental epigenetic modulators might be at work. It was surprising that FMPDs were more often unilateral suggesting the possibility of sampling variation or measurement error. Commonly, right and left retinas are more symmetrical in MPOD levels and spatial profiles [48, 49]. However, such asymmetry has been found in older population [50], history of central serous chorioretinopathy [51], and Starghardt’s disease [52].

EBAMD results agree with null genetic results from a similarly aged cohort evaluating SD-OCT retinal structure in pre-clinical AMD, against 17 SNP biomarkers [53]. The authors stated, “No consistent changes were observed in retinal structure at multiple locations that are associated with pre-clinical AMD, based on AMD genetic risk or with aging” [53], although neither macular pigment nor its foveal architecture were evaluated in this study.

In EBAMD, offspring of an AMD parent(s) presenting with a FMPD showed significantly reduced RBC serum concentrations of DHA compared to those without an FMPD. This was independent of other known FMPD risk factors in multivariate analysis, as well as general population risk factors. This seminal finding suggests that a low DHA concentration may play an age-independent role in the pathophysiology and hence vulnerability for AMD, in the offspring of an AMD parent(s). Indeed, offspring with FMPD were some 4 years younger, in agreement with authors who have found an increase in the RBC Omega-3 Index with age [54]. Our results reveal that offspring of an AMD parent(s), without any visible clinical signs of AMD, show an average yet insufficient Omega-3 Index [55]. It is possible that higher DHA may be required for the eye to mitigate retinal FMPD.

Although not statistically significant, FMPDs were associated with a higher L:Z ratio. It is known that in combination with the macular distribution of the 2 dietary xanthophylls (foveal zeaxanthin and parafoveal lutein), increased plasma zeaxanthin is significantly associated with reduced risk of AMD [56]. Indeed, it has been recently demonstrated that high dose (8 mg) zeaxanthin supplementation augments (‘normalizes’) both central MPOD and foveal visual scotomas in subjects presenting with atypical FMPD spatial profiles [40, 57, 58]. However, similar to Zeimer et al. [59], our data also suggests that the increased L:Z ratio does not create the FMPD but potentially amplifies it, which supports the hypothesis that lower serum Z (or higher L:Z ratio) only partially explains the presence of FMPD.

FMPD offspring exhibiting low (< 2.9 μg/dL) serum Z did not statistically differ compared to controls, however offspring of AMD patients presenting with low serum Z levels (~ 40%) had a significantly lower omega-3 index and significantly lower levels of DHA and HDL 2b. This suggests an important interaction between omega-3 fatty acids, lipoproteins and carotenoids such as zeaxanthin. It has been shown that DHA increases macular pigment in the central region, where zeaxanthin is the most prominent [60]. Furthermore, in a randomized, controlled trial, Johnson et al. [60] found that DHA facilitated accumulation of the carotenoid lutein in the blood and the macula, and that some of these effects may be due to alterations in lipoprotein profile by DHA. More work should be completed to study the possible interaction between omega-3 fatty acids, carotenoids and lipoproteins.

EBAMD also finds significant association between MPOD spatial profiles and central retinal thickness with individuals presenting with FMPD having thinner foveas. In Caucasians, a thicker central retina has been associated with significantly higher MPOD levels [61, 62], however, this was not evident from other reports [20, 21]. Studies have shown a significantly thinner central retina and wider foveas of healthy non-white patients compared to Caucasians [63–65]. Similar to heightened central dip prevalence, thinner retinas appear more comparable to ethnic populations that are known for increased prevalence of AMD [44, 45]. The relationship between the central- dip and AMD is still unclear because the mechanism of the formation of the central-dip is unclear. Mueller cells at the fovea contain macular pigment, and in many cases, the central dip is produced by the damage to Mueller cell cones induced by vitreous traction [66–68]. Furthermore, it is unknown whether the damage in Mueller cells, and not diminished pigment, becomes the risk factor for AMD development. The theme must be investigated further.

### EBAMD design, strengths, and limitations

A major limitation of this study is the lack of a control group of children who do not have a parent with AMD, as well as reliance on previous publications in which different instruments were used to measure macular pigment [19–22]. The issue of standardization using a newly introduced metric called MPOV (Macular Pigment Optical Volume), obtained by dual – wavelength autofluorescence, should be a major advance in confirming our results [69].

The utility of spectral reflectance to measure and visualize the foveal dip has been questioned and debated, especially in older patients [70]. Specifically, the major disadvantage of single-wave fundus reflectometry MPOD determination is the requirement for normal lens and retinal architecture [71]. Therefore, results may be unreliable in patients with advanced AMD or advanced cataracts. However, offspring with AMD and advanced cataract were excluded in EBAMD, rendering this limitation irrelevant or merely inconsequential.

There is a remote possibility that apparent MPOD foveal dips reflect differential absorption by other features of the lens or ocular media and the method used to assess MPOD (single wavelength autoflourescence) may not adequately correct for such features. However, again the patients in this study were relatively young, mitigating the confounding effects of lenticular or vitreoretinal pathology. Secondly, our 3D determination of a central MPOD dip in younger healthy offspring considered the relative change between the center and 2 degrees eccentricity, and the coefficient of repeatability was calculated in order to account for specular reflectance measurement variability.

Unlike heterochromatic flicker photometry (HFP), fundus reflectometry is an objective method of measurement and therefore may obtain MPOD estimates in special needs populations [37]. It has also proven to be repeatable and estimates of MPOD can be obtained in short duration [37, 38, 72]. Other strengths include objective assessment of carotenoid and fatty acid biomarkers instead of dietary questionnaires, and relatively equally sized cohorts.

## Conclusions

AMD offspring demonstrated non-protective macular pigment topography, often low (< 2.9 μg/dL) zeaxanthin, and significantly thinner foveas. Among all EBAMD factors and disease markers analyzed, % RBC membrane DHA was the magnanimous factor significant among 41% of AMD offspring with FMPD. EBAMD omega-3 RBC index data, a measure of long term essential fatty acid status, supports the importance of ‘essential fatty’ acids in AMD, and our contention that DHA is the predominant essential fatty acid.

Given the global AMD epidemic, Scripps EBAMD data are of clinical interest since participants were all AMD offspring lacking three of the major predisposing factors attributable to the MPOD foveal dip phenomenon: advanced age, smoking and an AMD clinical diagnosis. EBAMD data argues for further use of research and clinical instrumentation to measure MPOD distribution in AMD offspring, instead of peak MPOD screening, by the traditional but nonetheless entirely subjective HFP measure.

## Data Availability

Data described in the manuscript will be made available by the corresponding author (GAR) upon request.

## Abbreviations

AMD: Age-related macular degeneration
MP: Macular Pigment
RBC: Red blood cell
PUFA: Polyunsaturated fatty acid
MPOD: Macular pigment optical density
FMPD: Foveal macular pigment dip
DHA: Docosahexaenoic acid
EPA: Eicosapentaenoic acid
ALA: Alpha Lipoic Acid
DPA: Docosapentaenoic acid
cRT: Central retinal thickness
MTHFR: Methylenetetrahydrofolate reductase
